# Prevalence of methicillin-resistant *Staphylococcus aureus* colonization in HIV-infected patients in Barcelona, Spain: a cross-sectional study

**DOI:** 10.1186/s12879-015-0991-z

**Published:** 2015-06-26

**Authors:** Arkaitz Imaz, Mariana Camoez, Silvana Di Yacovo, Oriol Gasch, M Angeles Dominguez, Antonia Vila, Margarita Maso-Serra, Miquel Pujol, Daniel Podzamczer

**Affiliations:** HIV Unit, Infectious Diseases Department, Hospital Universitari de Bellvitge, L’Hospitalet de Llobregat, Barcelona, Spain; Microbiology Department, Hospital Universitari de Bellvitge, L’Hospitalet de Llobregat, Barcelona, Spain; Infectious Diseases Department, Corporació Sanitària Parc Taulí, Sabadell, Spain; Infectious Diseases Department, Hospital Universitari de Bellvitge, L’Hospitalet de Llobregat, Barcelona, Spain

**Keywords:** HIV, Methicillin-resistant *Staphylococcus aureus*, Colonization

## Abstract

**Background:**

Colonization by community-associated methicillin-resistant *Staphylococcus aureus* (CA-MRSA) has been found to be markedly more common in HIV-infected individuals in the USA. Studies evaluating the prevalence MRSA colonization in HIV-infected populations in Europe are scarce. The aim of this study was to investigate the prevalence of MRSA colonization in a cohort of HIV-infected patients in Barcelona, Spain.

**Methods:**

Nasal and pharyngeal *S. aureus* carriage was assessed in a random sample of 190 patients from an outpatient HIV clinic. Nasal and pharyngeal swab specimens were obtained for staphylococcal culture from 190 and 110 patients respectively. All MRSA isolates were screened for Panton-Valentine leukocidin (PVL) genes by PCR. Molecular characterization of MRSA isolates was performed by multilocus sequence typing. Data related to HIV infection, healthcare exposure, and previously described risk factors for MRSA were collected from medical records and a questionnaire administered to each patient.

**Results:**

The patients’ characteristics were as follows: male, 83 %; median (IQR) age, 45 (39–49) years; intravenous drug users, 39 %; men who have sex with men, 32 %; heterosexual, 26 %; CD4 count, 528/μL (IQR 351–740); on antiretroviral therapy, 96 %; and undetectable plasma viral load, 80 %. Sixty-five patients (34 %) were colonized by *S. aureus*. MRSA colonization was found in 1 % and 2 % of nasal and pharyngeal samples respectively. No PVL positive MRSA strains were detected and all the MRSA isolates belonged to typical hospital-acquired clones.

**Conclusions:**

Our data suggest that CA-MRSA colonization is not currently a problem in HIV-infected individuals in our area.

## Background

Methicillin-resistant *Staphylococcus aureus* (MRSA) has emerged as a causal agent of infection in individuals without risk factors for healthcare- associated MRSA acquisition that has been described as community-associated MRSA (CA-MRSA) [1]. CA-MRSA infections are usually distinguishable from their healthcare-related counterparts by epidemiological, molecular, and clinical features [2]. The incidence of the former is markedly increased in certain population groups such as HIV-infected patients, who have also been noted to have a higher risk of CA-MRSA colonization [3, 4]. In addition, HIV-infected individuals are frequently exposed to healthcare environments, and related factors such as prior hospitalization have also been linked to a higher rate of MRSA colonization in this population [5]. Nasal and extranasal MRSA colonization has been associated with an increased risk of subsequent infections in both HIV-negative and HIV-infected individuals [6, 7]. Additionally, colonization might represent a reservoir for MRSA transmission. As HIV-infected patients are commonly exposed to healthcare settings, it is important to assess the prevalence of colonization to determine the need for preventive and control measures, at least in hospital settings.

Most studies showing a high prevalence of MRSA colonization among HIV-infected individuals have been conducted in the USA. However, studies in certain US regions [8] and Europe [5, 9–12] have reported low prevalence.

Few European studies have evaluated MRSA colonization or infection in the HIV-infected population [5]. The aim of this study was to assess the prevalence of MRSA colonization in a cohort of HIV-infected patients in Spain.

## Methods

This is a cross-sectional study assessing *S. aureus* colonization in a random sample of 190 adults (>18 years) from a total of 1665 patients seen at an outpatient HIV clinic in Barcelona, Spain between June 2011 and June 2012. Patients were randomly selected using a statistical software package (IBM SPSS version 19.0, Chicago, IL). *S. aureus* colonization was investigated by taking nasal swabs from all 190 patients and pharyngeal swabs from 110 patients. Swabs were plated onto MRSA agar medium (MRSA Select; Bio-Rad Laboratories, Madrid, Spain) and coagulase-mannitol salt agar plates (BBL™ Coagulase Mannitol Agar; BD, Madrid, Spain), and also inoculated into staphylococcal enrichment broth (BBL brain-heart infusion; BD, plus 7 % NaCl). After 24 h of incubation at 35-37 °C, the broths were subcultured onto MRSA Select and coagulase-mannitol salt plates. All plates were incubated for 48 h and inspected daily for *S. aureus*–like colonies. *S. aureus* colonies were identified by latex agglutination (Pastorex Staph-plus; Bio-Rad Laboratories) and DNase production (DNase Test Agar; Difco, Fco. Soria Melguizo, Madrid, Spain). Antibiotic susceptibility testing, including cefoxitin, was performed by the disc-diffusion method following CLSI recommendations [13]. All MRSA isolates were screened for Panton-Valentine leukocidin (PVL) genes by PCR. Molecular characterization of MRSA isolates was performed by multilocus sequence typing and SCC*mec* characterization as described previously [14, 15].

HIV infection–related data and epidemiological characteristics previously described as community or healthcare-related risk factors for MRSA were collected from medical records and a questionnaire completed by each patient (Table 1) [1].

**Table 1:** Patient characteristics

	(*n* = 190)
Age, y (median, IQR)	45 (39–49)
Sex (male/female)	158/32 (83 %/17 %)
HIV risk factor	74 (39 %)
IDU	61 (32 %)
MSM	49 (26 %)
Heterosexual	6 (3 %)
Other/unknown	
AIDS	55 (29 %)
HCV	61 (32)
HBV	8 (4 %)
Current ART	182 (96 %)
CD4^+^ T cell count, cells/μL (median, IQR)	528 (351–740)
CD4^+^ T cell count, <200 cells/μL	19 (10 %)
HIV RNA, <40 copies/mL	153 (80 %)
Current use of TMP-SMX	15 (8 %)
Origin	Delete
Spain	161 (85%)
Europe	1 (0.5%)
South America	19 (10%)
North Africa	4 (2%)
Sub-Saharan Africa	4 (2%)
Asia	1 (0.5%)
Risk factors for HA-MRSA acquisition	
Antibiotic use in prior 12 months	70 (37 %)
Hospitalization in prior 12 months	32 (17 %)
Intravenous catheter use in prior 12 months	40 (21 %)
Surgical intervention in prior 12 months	19 (10 %)
Frequent visits (≥1 per week) to long-term care facilities	17 (9 %)
Multiple sexual partners in prior 12 months	17 (9 %)
History of sexually transmitted infection	17 (9 %)
Previous incarceration	29 (15 %)

Data are shown as number (%) of patients unless otherwise indicated.

IDU, injecting drug user; IQR, interquartile range; HBV, hepatitis B virus; HCV, hepatitis C virus; MSM: men who have sex with men; TMP-SMX: trimethoprim-sulfamethoxazole

CA-MRSA infection was defined according to the US Centers for Disease Control and Prevention 2000 criteria: 1) Diagnosis of MRSA in an outpatient setting or by a culture positive for MRSA within 48 hours of admission to hospital; 2) No medical history of MRSA infection or colonization; and 3) No medical history in the past year of: a) hospitalization; admission to a nursing home, skilled nursing facility, or hospice; c) dialysis; d) surgery; 4) permanent indwelling catheters or medical devices that pass through the skin into the body [1]. Since MRSA clones identified as typically CA-MRSA have distinguishing genetic features from hospital-acquired strains, such as SCC*mec* types IV and V and PVL expression, molecular characterization was also used to ascertain the possible origin of the strains (community vs healthcare environment) [2].

Continuous variables are reported as medians and interquartile range and categorical variables as numbers and percentages. The former were compared using the non-parametric Mann–Whitney U test while the latter were compared using the Χ^2^ or Fisher’s exact test, with a significance level of 0.05 (two-sided). A multivariate logistic regression model including demographic characteristics (age, gender, origin, HIV acquisition route), CD4+ T cell count, viral load, use of ART, healthcare-related related risk conditions (recent hospitalization or admission to a nursing home, recent surgical intervention, recent use of antibiotics), and other epidemiologic conditions (history of incarceration, history of sexually transmitted infections, multiple sexual partners) was used to identify independent risk factors for *S. aureus* and CA-MRSA colonization.

The study protocol was approved by the Clinical Investigation Ethics Committee of Bellvitge University Hospital and all patients gave written informed consent prior to participation.

## Results

The patients’ characteristics are summarized in Table 1. There were no significant statistical differences between patients with nasal and pharyngeal assessment (n = 110) and those with nasal assessment only (n = 80) (data not shown).

MRSA colonization was detected in 3 patients. Nasal colonization was observed in 2/190 individuals (1 %) and pharyngeal colonization in 2/110 individuals (2 %). Among patients with nasal and pharyngeal samples available, MRSA was present in both nasal and pharyngeal samples in 1 subject whilst the other patient was a pharyngeal carrier exclusively. Among those patients with MRSA colonization, two of them had risk factors for nosocomial acquisition. The two isolates were identified as ST146 and ST125, part of Clonal Complex 5 (CC5), and they both carried SCC*mec* IV (Table 2), which are indistinguishable features from the dominant hospital-acquired MRSA clone complex in our area [16]. The isolate belonging to the third patient was not available for molecular studies but antibiotic susceptibility and PVL results were available. Although the patient had no apparent risk factors for hospital acquisition, the antibiotic resistance pattern (resistance to erythromycin and ciprofloxacin) was identical to that of the dominant hospital-acquired MRSA lineage (Table 2).

**Table 2:** Characterization of MRSA isolates

Patient	Sample	SCC*mec*	MLST	PVL	Antibiotic resistance pattern
A	Pharyngeal	Type IV	ST146 (CC5)	Negative	Resistance to: erythromycin, clindamycin, tobramycin and ciprofloxacin
B	Nasal and Pharyngeal	Type IV	ST125 (CC5)	Negative	Resistance to: erythromycin, tobramycin and ciprofloxacin
C	Nasal	NA	NA	Negative	Resistance to: erythromycin, tobramycin and ciprofloxacin

CC, clonal complex; MLST, multilocus sequence typing; PVL, Panton–Valentine Leukocidin; SCC*mec*, staphylococcal cassette chromosome *mec*; ST, sequence type

Colonization by methicillin-susceptible *S. aureus* (MSSA) was documented in 62/190 nasal samples (32.6 %) and 3/110 pharyngeal samples (2.7 %), with only 1 in 3 patients found to be an exclusive MSSA pharyngeal carrier. None of the risk factors analyzed, including those related to demography, HIV acquisition route, HIV control, healthcare-related conditions, and other epidemiologic conditions, was statistically associated with MSSA colonization in either the univariate or multivariate analyses.

## Discussion

The prevalence of *S. aureus* colonization in our HIV-infected population is consistent with rates reported for the general population [17]. However, only 3 individuals (corresponding to 2/190 nasal samples and 2/110 pharyngeal samples) were colonized by MRSA and none of the isolates belonged to a typical CA-MRSA lineage. Our data contrast with most reports from the USA, where a significant association between HIV infection and MRSA colonization has been well documented [3–7]. A recent meta-analysis assessing MRSA colonization prevalence and risk factors in HIV-infected individuals reported that 6.9 % of the population studied were MRSA carriers and that this rate rose to 8.8 % when only studies from North America were included [5]. Although data on MRSA carriage in HIV-infected individuals in Europe are scarce, lower prevalence rates have been reported (0 % to 2.8 %) [5, 9–12], and the findings in our series are consistent with reports from other European studies.

Several US studies have described an association between CA-MRSA colonization and risk factors such as sexual behavior, substance abuse, incarceration, and area of residence [4, 6, 7]. These risk groups were represented in our sample (Table 1).

We recently reported a series of MRSA infection in HIV-infected patients in Spain in which we detected a higher risk for MRSA infection among individuals with poorly controlled HIV infection and immigrants (mostly South Americans) [18]. Nevertheless, the overall prevalence was still lower than that reported for the USA. The absence of CA-MRSA colonization in HIV-infected individuals in our area could partly explain the low rate of CA-MRSA infection in this population.

Our study is the first to assess the prevalence of MRSA colonization in HIV-infected patients in Spain. However, it has some limitations. Although our sample is representative of our cohort of HIV-infected patients, the number of individuals studied is lower than in other series. Additionally, while MRSA colonization of extranasal areas such as the buttocks and the perianal, inguinal and axillary regions has been well documented [4, 7, 19, 20], the only extranasal location studied in our case was the pharynx, and paired nasal and pharyngeal samples were only available for a subset of 110 patients. Thus, the rate of MRSA colonization in our cohort might have been underestimated. The importance of extranasal colonization has been demonstrated for both hospital- and community-acquired MRSA [4, 7, 19, 20]. Recent studies in the community setting have observed that nasal-only screening could miss up to 51 % of MRSA colonized individuals [4, 19]. In the subset of patients with both nasal and pharyngeal samples in our series, MRSA was detected in both samples in one patient while the other one was an exclusive pharyngeal carrier. None of the patients had exclusive nasal colonization in this subgroup. Although we do not discount the importance of extranasal colonization (the colonization rate would have been 50 % lower if our study had been limited to nasal samples), the rate of MRSA colonization in our cohort was low.

We were unable to genetically characterize one MRSA isolate. However, PVL expression, which is characteristic in CA-MRSA strains in our area, was not detected, and the antibiotic susceptibility pattern, while unspecific, was indistinguishable from the dominant hospital-acquired MRSA lineage. Thus, the molecular characterization results for the available isolates, the absence of PVL production, and the antibiotic resistance patterns detected suggested healthcare-related acquisition in all cases.

## Conclusions

Although nasal and/or pharyngeal *S. aureus* colonization was observed in one-third of HIV-infected patients in our cohort, the prevalence of MRSA carriage was low and colonization by typical CA-MRSA strains was not observed. Thus, MRSA colonization in the HIV-infected population in our area does not seem to be an epidemiological problem requiring specific control strategies to prevent cross-transmission and infection among HIV-infected individuals. More studies are needed to assess MRSA colonization rates in different European HIV-infected populations.
